# Gut Microbiome Development in Rock Pigeons: Effects of Food Restriction Early in Life

**DOI:** 10.3390/microorganisms13061191

**Published:** 2025-05-23

**Authors:** Maurine W. Dietz, Bin-Yan Hsu, Marco van der Velde, B. Irene Tieleman

**Affiliations:** 1 Groningen Institute for Evolutionary Life Sciences (GELIFES), University of Groningen, Nijenborgh 7, 9747 AG Groningen, The Netherlands; m.van.der.velde@rug.nl (M.v.d.V.); b.i.tieleman@rug.nl (B.I.T.); 2 Department of Life Science, Tunghai University, Taichung 407224, Taiwan; binyanhsu@gmail.com

**Keywords:** age, avian gut microbiome, gut microbiome development, host–microbiome interactions, rock pigeon, resource limitations

## Abstract

The developmental period is a critical phase in birds, influencing even lifetime reproductive success. The gut microbiome (GM) is important herein, affecting digestive capacity and immune function. Diet impacts the GM, but wild nestlings may experience resource limitations, which may also affect the GM. We investigated the effects of a week of food restriction early in life on the GM in captive rock pigeon nestlings (*Columba livia*). We sampled the GM at 0, 2, 4, 7, 8, 12, 20, 27, and 38 days and in foster parents. Alpha diversity varied only with age. However, differences in alpha diversity between nestlings and adults were larger during food restriction. Beta diversity varied with age, food treatment, and their interaction term. Four of the eleven major genera varied with age, while four others did not vary with age or food treatment. Major genera that contained potential pathogens (*Escherichia*-*Shigella* and *Clostridium sensu stricto 1*) were more abundant under food restriction. Food restriction thus affected GM development. The increase in alpha diversity and potential pathogens suggest that suppressed immune function may mediate the impact of food restriction on the GM. The effect diminished when food restriction was ended, suggesting that in wild nestlings, the impact of food restriction on the GM may be short-term.

## 1. Introduction

The developmental period is a vital period in the life of wild birds. Post-fledging survival, local recruitment rate and lifetime reproductive success are often associated with nestling growth patterns and fledgling mass [1,2]. In nature, many factors affect nestling growth and fledgling mass, such as food quality and quantity, weather conditions, timing within the breeding season (early vs. late broods), and sibling competition [1,2,3,4]. Also the development of the nestlings’ physiological maturity, for example, the digestive capacity to utilize resources, plays an important role [4,5]. The digestive capacity of a nestling is determined by the gut’s physiological maturity and by the gut microbiome, which provides essential digestive functions such as the breakdown of nutrients and the synthesis of vitamins and amino acids [6,7]. Moreover, the gut microbiome is crucial for postnatal development by releasing bacterial products essential for the development of organs, immune system, metabolic stimulation, social behavior, and cognition [8,9,10,11]. In birds, the establishment of the gut microbiome may already start in the egg, as shown in captive rock pigeons, homing pigeons (*Columba livia*), and chickens (*Gallus domesticus* [12,13,14]), yet at hatching, the gut microbiome is still an unstable community that develops over time into a complex, established community. The development of the gut microbiome is affected by intricate interactions between food resources, the gut, and systemic immune function, vertical transmission of microbes by parents, and the influx of microbes living on food, and in the nest and habitat environment [6,15]. In addition, differences in initial colonization of the neonatal gut may trigger different successional patterns in microbiome composition [16,17]. Although recently gut microbiome development in birds has received more attention [12,13,14,18,19,20,21,22], how the gut microbiome is acquired and what factors affect gut microbiome development in birds are still largely unknown.

Key factors hypothesized to affect gut microbe community composition are diet composition and food quantity [6,15,23]. In nature, nestling diet may vary with habitat, nestling age, and timing in the breeding season. Weather conditions may affect food quality and quantity, as well as the feeding frequency of nestlings. During adverse weather conditions, nestlings of wild birds commonly experience periods of resource limitations [1,24,25]. Resource limitations of nestlings may result in limited resources for gut microbes, which may potentially influence gut microbiome development. Gut microbes are regularly exposed to limited resources, e.g., when hosts consume food only during daytime or nighttime. Some gut bacteria are more resistant to limited resources than others. For example, Bacteroidetes species utilize specific energy-saving protein synthesis pathways under carbohydrate limitations, switch their metabolism under polysaccharide limitations toward degradation of host glycans, and can metabolize protein and fat provided by the intestinal epithelium [26,27]. Resistance to limited resources may favor the survival of such specific microbes when resources are limited for a longer period, and may thereby affect the gut microbiome development.

Given the regular occurrence of periods of resource limitations in the field, we investigated the effects of a week of food restriction early in life on the developmental trajectory of the gut microbiome in captive rock pigeon nestlings. Rock pigeons are the wild-type pigeon from which domestic pigeon breeds descend. They lay clutches of two eggs, and both parents care for the nestlings [28]. During the first week of life, nestlings are fed a regurgitated nutrient-rich crop secretion called crop milk that consists of protein (~60%), fat (~32–36%), immunoglobulins, enzymes, carotenoids, growth factors, minerals, and parental microbes [14,29,30,31]. At the end of the first week, parents start to include semi-digested food in the nestlings’ diet, and from ca. day 14 onward, nestlings receive only semi-digested food [28,30]. We restricted the amount of food that the nestlings received until day 8 (day 0 is day of hatching) by restricting the amount and quality of food provided to their foster parents, which resulted in lower parental crop milk production. Previous experiments showed that this is an effective way to food-restrict rock pigeon nestlings, as indicated by lower nestling body masses [32]. Parental challenges may have different effects on crop milk components. For example, parental feral pigeons (*Columba livia*) immune-challenged by keyhole limpet haemocyanin (KLH) injections did not change anti-KLH antibody levels in their crop milk, while natural antibody levels increased [29]. We did not collect parental crop milk and do not know if crop milk composition changed under food restriction.

To determine the effects of food restriction early in life on gut microbiome development, we focus on the bacterial gut community, which represents an important and well-known component of the gut microbiome. We collected the first feces of rock pigeon hatchlings and cloacal swabs from nestlings at days 2, 4, 7, 8, 12, 20, 27, and 38 and from adults. We expect that 1) food restriction may increase the competition between gut microbes, favoring bacterial taxa better adapted to survive under limited resources, resulting in a lower diversity of the gut microbiome. An earlier study found that food restriction suppressed aspects of systemic immune function in rock pigeon nestlings [32]. If food restriction suppresses the immune function, we expect 2) that this may result in less control of the gut microbiome by the impaired immune system of the nestling, which may lead to higher microbe diversity and potentially also a higher pathogen presence. When the nestlings return to normal food conditions, we expect that 3) the potential gut microbiome differences between nestlings under normal and food-restricted conditions would diminish when food quality and quantity largely determine gut microbiome composition, but 4) may be maintained if the initial colonization differed between food treatments and that this strongly impacts gut microbiome development.

## 2. Materials and Methods

### 2.1. Animals and Sample Size

This study belonged to a larger experiment focusing on the maternal effects of yolk testosterone concentrations on nestling development in relation to food restriction. The larger experiment started in late March 2013 at the Groningen Institute for Evolutionary Life Sciences of the University of Groningen (53°14.579′ N, 6°32.271′ E). The rock pigeon pairs were housed in outdoor aviaries (4.0 m × 1.7 m × 2.2 m; one pair per aviary) and provided with a nest box (60 cm × 50 cm × 36 cm). For more details, see [13].

Spring 2013 was very cold, which had especially a negative impact on the survival of food-restricted nestlings. Because we did not aim to investigate the effects of very poor conditions on the development of gut microbiota, we only analyzed the data of food-restricted broods where both nestlings survived until fledging (three broods sampled in June–August). For a balanced design, we limited the analyses of broods receiving the normal food treatment to three randomly selected broods in which both nestlings fledged, and these were sampled in June–August. Thus, we analyzed in total the samples of 6 of the 21 broods that started in June–July (29%), i.e., in total 108 nestling samples, and 11 adult samples (one male died prior to cloacal sampling: see Section 2.5).

### 2.2. Formation of Experimental Clutches

Every morning, we checked the nest boxes for freshly laid eggs, which were marked and collected. We replaced these eggs with dummy eggs to ensure that the parents would continue laying/incubation. We used only first-laid eggs to form experimental clutches, and stored these at 12–16 °C and 40–50% RH (maximum 3 days), until we could assign the batch to experimental clutches with matching egg mass and laying date. For each experimental clutch, we injected the yolk of one egg with testosterone (T-egg, see Supplementary Materials) and the yolk of the other egg with the vehicle, sesame oil (C-egg). Testosterone injections increased yolk testosterone levels to that naturally occurring in second-laid eggs: testosterone levels are on average 5.18 pg/mg yolk in first-laid eggs and 18.99 pg/mg yolk in second-laid eggs [33]. Injections in eggs are applied in research on birds and reptiles aiming to manipulate yolk hormone levels or to sample egg contents while maintaining good hatchability [34,35]. The experimental clutch was placed with an unrelated foster pair.

### 2.3. Collection of First Feces and Formation of Experimental Broods

**Figure 1:**
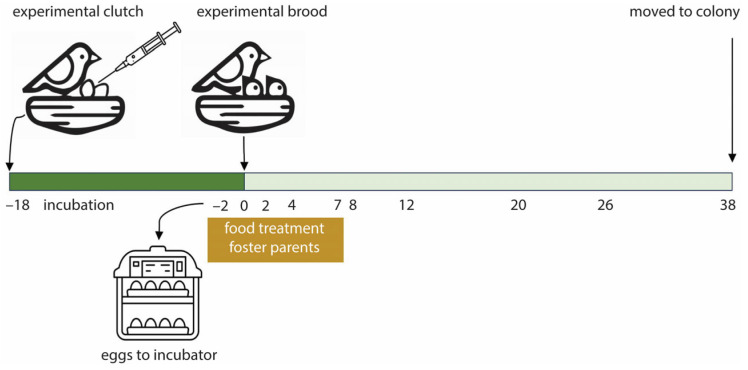
Overview of the experimental setup. The original clutch of a pair was replaced by an experimental clutch containing an unrelated T- and C-egg matching in mass and laying date. Two days before the expected hatching date, the eggs were replaced by dummy eggs, moved to an incubator, and the food treatment of the foster parents started. Upon hatching, experimental broods were formed of hatchlings differing in yolk testosterone treatment, but matching in body mass, sex, and hatching time. The nestling gut microbiome was sampled at the days indicated. At day 0, feces were collected and cloacal swabs were taken of older nestlings. At fledging, day 38 post-hatch, the nestlings were moved to the colony.

To record hatching time and collect the hatchlings’ first feces, we replaced the eggs with dummy eggs on day 16 of incubation and moved the eggs to an incubator set at 37.5 °C and >75% RH (Figure 1; overall incubation period ~18.5 days; [28]). We checked the incubator three times a day (9:00, 13:00, and 17:00) for hatched nestlings. If hatching signs were present at 17:00, we checked the incubator again at 21:00. Hatchlings generally deposit their first feces immediately after hatching in the eggshell. We collected the first feces with a sterile viscose swab (Copan) and placed them into a sterilized vial (1.5 mL, including the tip of the swab). Feces found outside the eggshell were not collected. To prevent desiccation, a drop of sterilized phosphate-buffered saline (PBS) was added to the samples before storing them at −20 °C until analysis. The hatchling was weighed (±1 g), tarsus length was determined (±0.1 mm), and a small blood sample (<75 µL) was taken from the medial metatarsal vein to determine sex [36]. For the larger experiment, we created experimental broods with two hatchlings matching in sex, body mass, and hatching time, but differing in yolk testosterone treatment. Hence, the hatchlings were reshuffled according to these criteria and returned to new, unrelated foster parents. The day of hatching was regarded as day 0 of the experiment.

### 2.4. Food Treatment

The normal diet consisted of ad libitum standard short beak pigeon food (Kasper Faunafood 671220, Woerden, The Netherlands) and turtle dove food (Kasper Faunafood 672120, Woerden, The Netherlands ), water, and a mixture of small stones and pigeon grit. Foster pairs were randomly assigned to a food treatment. The food restriction treatment started 2 days before the expected hatching date, when the eggs were moved to the incubator (Figure 1). The pairs received a limited amount of grain mixtures (43 g per pair) with lower protein content [37], ad libitum water, and a mixture of small stones and pigeon grit. The 43 g food was based on the average daily consumption of a not reproducing pair, 33 g [38], plus 5 g food per nestling per day. Food restriction lasted until the nestlings were 8 days old. From day 8 onwards, food-restricted parents again received the normal ad libitum diet. The normal food group continued with the ad libitum diet, water, and small stones and grit when their eggs were moved to the incubator.

At fledging (day 38), the nestlings were moved to our rock pigeon colony so that the foster parents could start another brood. Note that free-living rock pigeons breed year-round when conditions permit [28].

### 2.5. Nestling Development and Cloacal Swabs

We took cloacal swabs and determined nestling body mass and tarsus length every two days until day 14, then every 3 days until day 26, and at fledging (day 38; Figure 1), except for day 7, when body mass and tarsus length were not determined. To collect cloacal swabs, we inserted the tip of a sterile viscose swab fully in the cloaca, rotated it for ca. 10 s, and stored the tip of the swabs in sterilized 1.5 mL vials. A drop of sterilized PBS was added before storage at −20 °C until analysis. Because rock pigeons are sensitive to disturbance during breeding, we collected cloacal swabs of the foster parents at the end of the experiment in August, shortly after the last nestling samples were taken. Due to this, we could not sample one parent (male, food-restricted conditions) that died before the end of the experiment.

### 2.6. DNA Isolation and 16s rRNA Gene Amplicon Sequencing

The samples were randomized before DNA extraction (PowerSoil^®^ DNA kit, MoBio, Carlsbad, CA, USA; see Supplementary Materials) and the extracted DNA at −20 °C stored until further use. We quantified DNA concentrations (Quant-it PicoGreen dsDNA kit, Molecular Probes, Invitrogen, Eugene, OR, USA) to normalize DNA concentrations in the PCR to 1 ng template DNA per 25 µL reaction. We randomized the samples again before amplifying the V4–V5 region of the 16S rRNA gene in triplicate using the primers 515F and 926R [39,40]; see Supplementary Materials). The triplicate PCRs were pooled, purified (QIAquick gel extraction Kit, Qiagen GmbH, Hilden, Germany) and sent to GenoToul (INRA, Toulouse, France) for library preparations and Illumina sequencing using 2 × 250 bp v2 chemistry (114 pigeon samples, a negative control swab, and 4 negative PCR controls; DNA concentrations of 5 pigeon samples were too low to be sequenced). At GenoToul, the sequence reads were demultiplexed and quality-filtered using the default settings in QIIME1.

### 2.7. Sequence Data Processing

The raw sequence data were processed following the standard QIIME2 protocol (v2018.2; [41]). Using the DADA2 (v2018.2) pipeline, we trimmed the primers, truncated the forward and reverse reads to 250 bp and 190 bp, respectively, merged the forward and reverse reads based on quality plots (at least 25 bp overlap), and removed chimera. We built the taxonomy table using the Silva v132 reference database [42,43]. *Archaea*, chloroplasts, mitochondria, vertebrates, and one unknown kingdom were filtered from the data. The end products, an amplicon sequence variants (ASV) table, and the phylogenetic tree were processed in R (v4.2.2; [44]) using Phyloseq (v1.44.0; [45]) and vegan (v2.6-4; [46]). At this stage, the data included 1678 taxa, and the total number of sequence reads was 3,571,875, with the rock pigeon samples counts ranging between 1137 and 93,277 reads and the negative control samples between 74 and 1472 reads.

### 2.8. Statistical Analyses

#### 2.8.1. Effects of Food Restriction on Nestling Growth

We used linear mixed models (LMMs, nlme v3.1-148; [47]) to identify the correlations between nestling body mass or tarsus length and age, food, and testosterone treatment, with interaction terms age*food treatment and testosterone treatment*food treatment (fixed factors), and individual nestling nested within aviary (random factors). The interaction terms were included because food restriction was limited to the first week of life only, and food treatment and testosterone treatment may interact. We used a stepwise backward exclusion of nonsignificant fixed factors. At each step of the analysis, we visually inspected model validation plots to confirm model assumptions. For the final models we tested if the aviary contributed significantly to the model (ANOVA), which was never the case. Testosterone treatment and testosterone treatment*food treatment never contributed significantly to the final models. We calculated the marginal and conditional *R*^2^ for the final models using the r.squared GLMM function of the R package *MuMIn* v.1.47.5 [48]. We used all available body mass and tarsus length data for these analyses, i.e., the metadata prior to rarefying, and thus the sample size was 6 nestlings (3 nests) per age per food treatment. Note that body mass and size were not determined at day 7.

#### 2.8.2. Contamination and Rarefication

Using the prevalence method of Decontam (v1.20.0; [49]), we identified eight contaminant ASVs and removed these from the data. Thereafter, we removed the negative control samples from the dataset, which eliminated another 20 ASVs from the data. At this point, the data included 1650 taxa divided over 114 samples, with a total of 3,567,415 reads.

Because we sampled throughout nestling development, we had a large variation in read points per sample, and hence we rarefied the data, the most commonly used method to transform sequence data prior to analyses [50,51]. Richness rarefaction curves leveled off around 4000 reads (Figure S1), so we rarefied the data to 4980 reads, which was close to the sample with the lowest number reads above 4000 reads (4981 reads). Rarefying excluded five samples from the dataset: two samples of nestlings of 0 days old (one C-egg, one T-egg, both normal food conditions), one sample of a 26-day-old nestling (C-egg, normal food), and two adults (one male, one female). Thereafter, 1119 taxa were left divided over 109 samples: 100 nestling and 9 adult samples. We used the rarefied data in the analyses described below.

#### 2.8.3. Effects of Food Restriction on Alpha Diversity

Similar to the analysis of nestling parameters, we used LMMs to identify correlations between alpha-diversity indices of nestlings (richness, Shannon index, Chao1 and Faith’s phylogenetic diversity) and age, age^2^, food and testosterone treatments, standardized body mass, the interaction terms age*food treatment and testosterone treatment*food treatment (fixed factors), and individual bird nested within aviary (random factors). Age^2^ was included because the alpha-diversity indices varied non-linearly with age. Body mass was included to test if alpha diversity consistently differed between nestlings that were relatively heavy or light. Because body mass is strongly correlated with food treatment, age and age^2^, we standardized body mass per age by a z-transformation. We excluded the foster parents from the analyses because of the large and variable age difference between them and the oldest nestlings.

To determine if the alpha diversity of the nestlings’ gut microbiome developed towards that of an adult gut microbiome, we calculated the differences between nestling and adult alpha diversity and tested with LMMs if these differences varied with the fixed factors nestling age, food and testosterone treatment, the interaction terms age*food treatment and testosterone treatment*food treatment, and individual nestling nested within aviary as random factors.

Testosterone treatment, testosterone treatment*food treatment, body mass and aviary never contributed significantly to the final models.

#### 2.8.4. Effects of Food Restriction on Community Composition

We assessed the bacterial community composition (beta diversity) by examining the taxonomic similarities between age and food treatment using the Jaccard similarity index (community membership: presence/absence), Bray–Curtis dissimilarities (community structure: presence/absence and abundance matrix), and by examining the phylogenetic similarities between age and food treatment using unweighted (community membership: presence/absence table) and weighted UniFrac distances (community structure: presence–absence–abundance matrix; [52]). Because PERMANOVA assumes categorical variables, we could combine nestlings and foster parents in the analyses. We tested with a principal coordinate ordination analysis (PcoA) if community clustering and group dispersion differed between age, food treatment, and interaction term age*food treatment, by modeling beta diversity (dis)similarities and distances from an ASV-level table using PERMANOVA with 10,000 permutations (ADONIS2 function of vegan [53,54]). Testosterone was excluded from the analyses because foster parents did not receive this treatment. PERMANOVA is sensitive to the factor order in the model, and hence we reran the models in all possible factor orders. We evaluated the degree of within-group dispersions (permutest) for age and food using the betadisper function [55]. These were nonsignificant apart from weighted UniFrac distances, where the permutest for age was significant, indicating that differences found may be due to differences in age group dispersions.

#### 2.8.5. Effects of Food Restriction on Taxonomic Composition

We used the same LMM procedure as described for alpha diversity to examine the associations within nestlings between the relative abundance of the most abundant phyla (>5%) and genera (>2%) and age, food and testosterone treatments and standardized body mass. Because the data were inflated with zero abundance, the taxon proportions were logit-transformed as log[(*p* + *e*)/(1 − *p* + *e*)] before running the LMMs, where *p* is the proportion of a taxon in a given sample and *e* the lowest proportion (among samples) for that taxon excluding zero [56]. Testosterone treatment, testosterone treatment*food treatment, body mass and aviary never contributed significantly to the final models.

We used the R package specificity (version 0.1.16.9; [57]) to determine which ASVs were specific to a limited range of ages in nestlings per food treatment group. Following the guidelines, we set the number of simulations at 1000 and excluded ASVs with a prevalence < 10 from the analyses, which reduced the number of ASVs to 67 for nestlings whose foster parents received normal food treatment and to 74 for nestlings whose foster parents were food-restricted. All ASVs specific for an age range showed a peak in proportions, and we visually determined the age range over which the proportions peaked.

#### 2.8.6. Effects of Food Restriction on Potential Pathogens

We explored the effects of food restriction on the presence of potential pathogens by examining the presence and abundance of five bacterial pathogens that are common in homing pigeons, the domesticated form of rock pigeons, namely *Salmonella* (especially *S. typhynusium*) and *Escherichia coli*, which both always cause disease in homing pigeons, and *Straptococcus galloticus*, *Chlamydophila psittaci*, and *Enterococcus*, which are facultative pathogens in homing pigeons [58]. Note that the SILVA database assigns taxa up to genus level, thus rarely to species level. Hence, although we did look for the specific species, we also included genus level in the analyses.

## 3. Results

### 3.1. Effects of Food Restriction on Nestling Development

**Figure 2:**
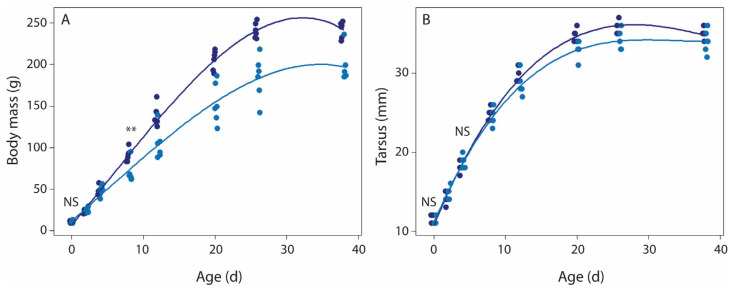
The effects of food restriction on body mass (**A**) and tarsus length (**B**) development in rock pigeon nestlings. LLM analysis showed that development of both body mass and tarsus varied with age, age^2^, and age*food treatment (Table S2). T-tests showed that hatchlings of both food treatments did not differ in body mass and tarsus (NS), while after the food restriction period, day 8 nestlings of the food-restricted group were lighter (**, *p* < 0.01), but not smaller (NS, tarsus length) than nestlings of the normal food treatment group. Normal food treatment, dark blue symbols; food-restricted treatment, light blue symbols. Sample size per age per treatment group: 6 nestlings (3 nests).

Hatchling body mass and tarsus length did not differ between food treatment groups (Student’s *t*-test, body mass *t* = 0.00, *df* = 9.22, *p* = 1.00; tarsus length *t* = 1.12, *df* = 10 *p* = 0.29; Table S1). At the end of the food treatment period (day 8), food-restricted nestlings (69.5 ± 12.7 g) were much lighter than normal food nestlings (90.5 ± 7.9 g; *t* = 3.44, *df* = 8.34, *p* = 0.008), confirming that the food restriction of the parents indeed resulted in less food for nestlings, yet tarsus length did not differ between food treatments at day 8 (24.8 ± 0.8 mm vs. 24.2 ± 1.2 mm, normal vs. food restriction, respectively; *t* = 1.17, *df* = 8.54, *p* = 0.27). LMM analyses showed that the body mass difference was maintained over the subsequent development, and hence initially food-restricted nestlings were considerably lighter at fledging (Figure 2A, Table S2). Overall tarsus length development was affected by food treatment (Figure 2B, Table S2), but the differences between treatments were small.

### 3.2. Food Restriction Affects Alpha Diversity

**Figure 3:**
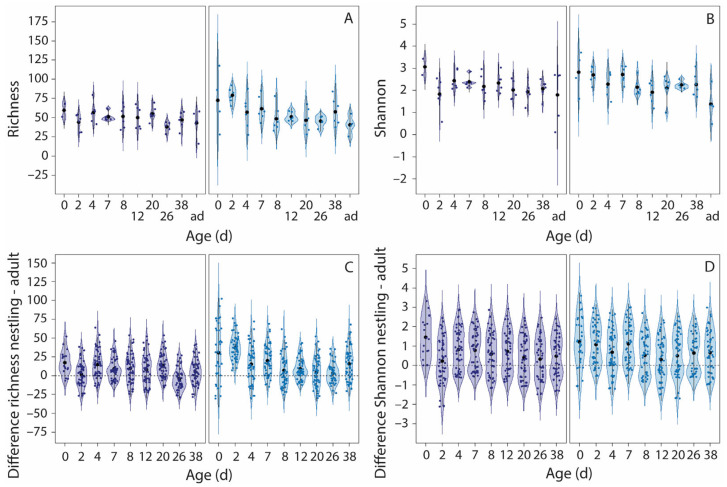
The effects of food restriction on the development of richness and Shannon index. Presented are the variations in richness (**A**) and Shannon index (**B**) with age and the variations in the differences in richness (**C**) and Shannon index (**D**) between nestlings and all adults (ad) per age. Left panels, normal food treatment (dark blue violins), right panels, food restriction treatment (light blue violins). Black symbols indicate the mean, the blue symbols are the raw data. The dashed line in the bottom panels indicates no difference between nestlings and adults. Note that the adults are included in panels A and B, but not in the LMMs, where age is included as a continuous variable. Sample sizes, panels A and B: per age per treatment group 6 nestlings (3 nests), except for day 0 (2 normal food nestlings and 3 food-restricted nestlings), and day 26 (5 nestlings per age per treatment group). Sample sizes, panels C and D: for each nestling presented in A and B, the differences in alpha diversity with 9 adults are given, thus 6 × 9 = 54 data points per age and treatment group, except for day 0 (18 data points for normal food nestlings, and 27 data points for food-restricted nestlings) and day 26 (45 data points per age per treatment group).

All alpha-diversity indices varied nonlinearly with nestling age, with age and age^2^ explaining 9–11% of the variation (Figure 3A,B and Figure S2A,B, Table 1). Alpha diversity was highest in very young nestlings and decreased during the first week after hatching, whereafter levels stabilized. Developmental trajectories of alpha diversity were not affected by food restriction.

At early ages, alpha diversity was higher in nestlings than in adults, but from day 8 onwards, nestling alpha diversity was comparable to that of adults, except for the Shannon index, which remained higher in nestlings (Figure 3C,D and Figure S2C,D, Table S3). Alpha diversity of fledglings (day 38) and adults was similar (student *t*-test, all *p* > 0.09). Food restriction resulted in more pronounced differences between nestlings and adults at early ages than under normal food conditions, except for the Shannon index, where food treatment did not affect the variation.

### 3.3. Food Restriction Affects Community Composition

**Figure 4:**
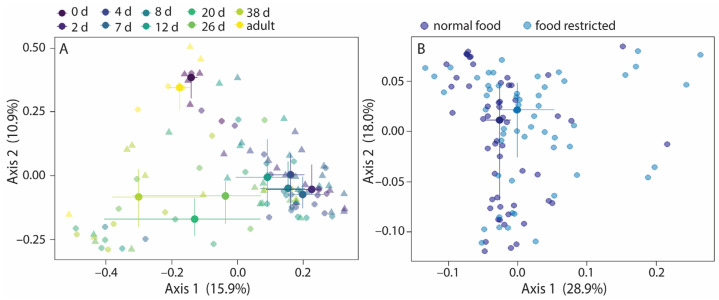
Variation in beta diversity with age and food treatment in nestlings and adults. The Bray–Curtis dissimilarities (**A**) varied with age, food treatment, and the interaction term age*food treatment. For weighted UniFrac distances, only the food treatment groups are indicated; age was excluded from the model because the permutest was significant (**B**). Large symbols present medians, the error bars the 25% and 75% quantiles. Transparent symbols present the underlying data. Symbol shapes, panel A: circles, normal food treatment; triangles, food restriction treatment. Sample sizes: per age per treatment group 6 nestlings (3 nests), except for day 0 (2 normal food nestlings and 3 food-restricted nestlings), and day 26 (5 nestlings per age per treatment group); and 9 adults.

Jaccard similarities and Bray–Curtis dissimilarities varied with age, food treatment, and their interaction term within nestlings and adults, while unweighted UniFrac distances varied with age and food treatments (Figure 4A and Figure S3A,B). Age explained 15.3–25.6% of the variation (Table 2), while food treatment explained 1.4–1.9% of the variation, suggesting that the direct effect of food restriction on the community composition was small. The interaction term age*food treatment explained 8.7–8.8% of the variation, indicating that Jaccard and Bray–Curtis variation with age was affected by food restriction. Post hoc analyses for age showed that the community composition of day 0 and day 2 old nestlings especially and adults differed from that of other nestling ages (Figure S4). The permutest for age was significant for weighted UniFrac distances, and thus the age effect could be due to variations in group dispersion for weighted UniFrac. Therefore, the model was rerun with only food treatment as explanatory factor. Food treatment was significant, but explained only 2.9% of the variation, just as for the other beta diversity values (Figure 4B, Table 2).

Since the results indicated that the effect of food restriction was largest during the period when it was given, we repeated the analyses for 0- to 7-day-old nestlings. Indeed, the direct contribution of food treatment to the variation in beta diversity more than doubled to 4.0–6.6% (Table S4), while the indirect contribution of food treatment via the interaction term with age remained comparable, 7.5–8.7% (Table S4).

### 3.4. Effects of Food Restriction on the Taxonomic Composition

The three most abundant phyla (relative abundance >5%; nestlings and adults combined) were Firmicutes (50.3% ± 28.0%, mean ± SD), Actinobacteria (31.8% ± 23.0%) and Proteobacteria (14.1% ± 8.5%). Tenericutes (2.8% ± 15.3%) reached relatively high abundance also, but only in adults. Relative abundance of the other phyla was <1% (Figure S5). Within nestlings, the logit-transformed proportions of Firmicutes, Actinobacteria, and Proteobacteria did not vary with age, food treatment or standardized body mass (LMMs, all *p* > 0.05).

**Figure 5:**
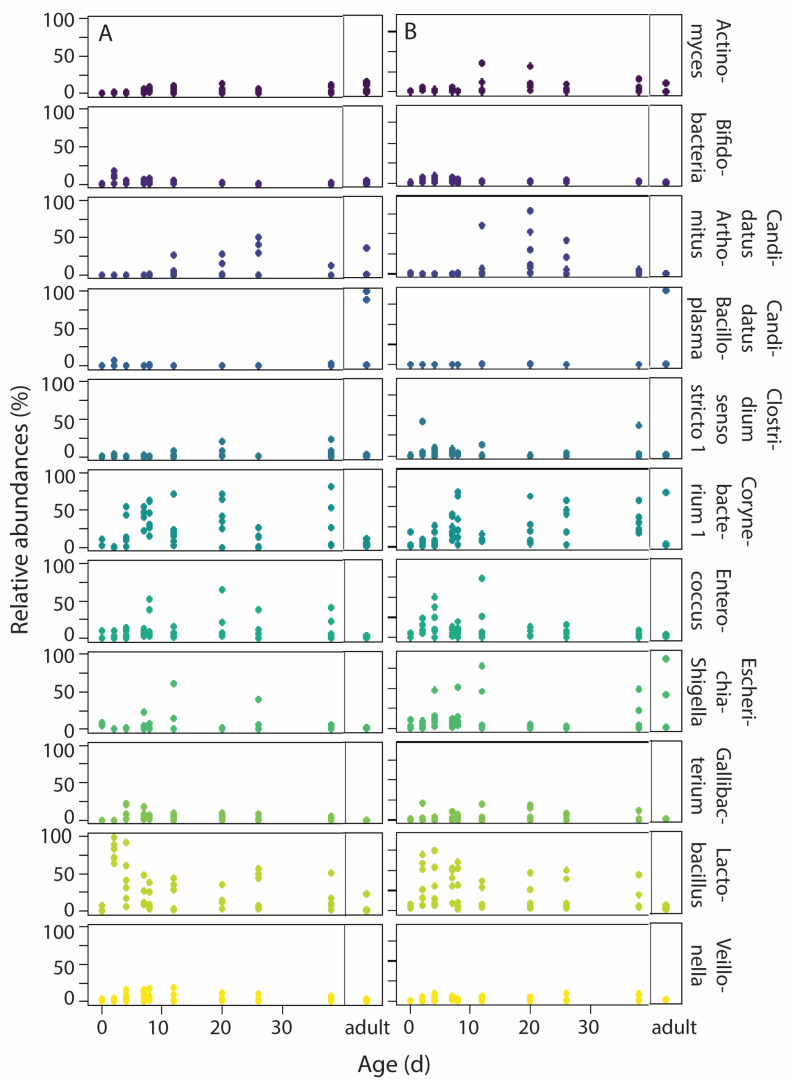
Variation in relative abundance of the most abundant genera (>2%) with age under normal food (**A**) and food-restriction conditions (**B**). Adults are presented in separate panels per food treatment. Sample sizes: per age per treatment group 6 nestlings (3 nests), except for day 0 (2 normal food nestlings and 3 food-restricted nestlings), and day 26 (5 nestlings per age per treatment group); and 9 adults. Colors present the different genera; genera names are given at the right of panel B.

Eleven genera had high abundance (>2%, Figure 5, Table S5), of which *Lactobacillus* (Firmicutes, 24.7% ± 24.4%) and *Corynebacterium 1* (Actinobacteria, 21.6% ± 1.5%) had the highest abundance. Logit-transformed proportions of *Corynebacterium 1* varied with nestling age (*p* = 0.001) and age^2^ (*p* = 0.018, Table 3): proportions were initially low, but high from approximately day 7 onwards. Logit-transformed proportions of *Lactobacillus* did not vary with age or any other factor (LMMs, all *p* > 0.05), and were high throughout the nestling developmental period. The logit-transformed proportions of *Enterococcus*, *Veillonella* (Firmicutes) and *Gallibacterium* (Proteobacteria) did not vary with any factor either (LMMs, all *p* > 0.05). The distribution of the LMM model for *Candidatus Bacilloplasma* (Tenericutes) was not normal and the LMM could not be conducted. The logit-transformed proportions of the other abundant genera varied with nestling age: with age and age^2^ in *Actinomyces* (Actinobacteria) and *Candidatus-Arthromitus* (Firmicutes), and with age in *Bifidobacterium* (Actinobacteria). Just as for *Corynebacterium 1,* relative abundance of *Actinomyces* and *Candidatus-Arthromitus* were higher after the first week of life. In contrast, relative abundance of *Bifidobacterium* was highest during the first week of life. Logit-transformed proportions of *Clostridium sensu stricto 1* (Firmicutes) varied with age*food, being higher in food-restricted nestlings. Lastly, *Escherichia-Shigella* (Proteobacteria) logit-transformed proportions were higher under food restriction, but did not vary with age (Table 3).

The proportions of 10 ASVs, divided over five known and three unknown genera, were specific for an age range under normal food conditions, while the proportions of 12 ASVs, divided over eight known and four unknown genera, were specific for an age range under food-restriction conditions (Table S6). For both food treatments, the genera *Actinomyces*, *Bifidobacterium* and *Corynebacterium1* were specific for age and belonged to the most abundant genera (Table S5), while *Gallibacterium* was an abundant genus specific for age under normal food conditions, and *Escherichia-Shigellla* was an abundant genus specific for age under food-restriction conditions. Most of the ASVs specific for an age range belonged to Actinobacteria (seven ASVs for both normal food and food-restricted nestlings), and four of these Actinobacteria ASVs were age-specific in both food treatment groups (Table S6). Under normal food conditions, the proportions of most Actinobacteria ASVs peaked from day 2 until 12 (Figure S6), but under food restriction, the proportions of Actinobacteria ASVs peaked mostly over a shorter period, ranging from days 2–4, 4–7, to 2–12 (Figure S7). A similar pattern was seen for the other ASVs (Firmicutes, two and four ASVs, respectively; Proteobacteria, one ASV for both food treatments), where peaks in ASV proportions lasted longer under normal food conditions than under food restriction.

### 3.5. Effects of Food Restriction on Potential Pathogens

The genera of two of the five homing pigeons’ common pathogens were not present in any sample, namely *Salmonella* (*S. typhinurium*) and *Chlamydophila* (*C. psittata*). We did not detect the specific pathogen species *Streptococcus galloyticus* and *Escherichia coli*, but their genera were present: *Streptococcus* (13 ASVs) and the clustered *Escherichia-Shigella* genus (14 ASVs). Lastly, the *Enterococcus* genus (28 ASVs) was also found. *Enterococcus* and *Escherichia-Shigella* belonged to the most abundant genera (7.7% and 9.8%, respectively), while an *Escherichia-Shigella* ASV was specific for an age range under food restriction (Table S6). Also the logit-transformed proportions of *Escherigia-Shigella* were higher under food restriction (Table 3), while the logit-transformed proportions of *Enterococcus* did not vary with any factor (LMM, *p* > 0.05). The relative abundance of the *Streptococcus* genus was low 0.24%. The initial model of the LMM of the logit-transformed proportions was not normally distributed. Visual inspection of the variation in proportion with age and food (Figure S8) did not suggest that food restriction affected *Streptococcus* abundance.

## 4. Discussion

Parental food restriction resulted in food restriction of nestlings, as shown by the nestlings’ lower body masses. Food restriction had more subtle effects on gut microbiome development than expected, and these effects occurred mainly during the first week of life, the period when the nestlings were food-restricted. Food restriction had little effect on five common pigeon pathogens: only *Escherichia-Shigella* was more abundant under food restriction (note that two pathogens were not present). Age was the major factor influencing gut microbiome development, with most changes occurring during the first week of life.

### 4.1. Effects of Food Restriction on Alpha and Beta Diversity

Although food treatment did not affect the developmental patterns of alpha diversity directly, food restriction did result in larger differences between nestling and adult alpha diversity at early ages—alpha diversity being higher in nestlings than in adults. This suggests that in very young nestlings, food restriction increases alpha diversity, which may be mediated by a suppressed immune function due to food restriction (contra expectation 1, pro expectation 2 [32]). Under both food treatments, alpha diversity stabilized at levels comparable to adults after the first week of life, indicating that differences due to food treatment diminished quickly when the food treatment was ended (pro expectation 3, contra expectation 4).

Alpha diversity of rock pigeon nestlings was variable during the first week of life: it peaked at day 2 and decreased during the first week after hatching to stabilize thereafter. Such a developmental trajectory has been found in other bird species, for example, in nestlings of homing pigeons, zebra finch (*Taenopygia guttata*), great tit (*Parus major*), dunlin (*Calidris pusilla*), and red pharalope (*Pharalopus fulicarius*) [14,18,19,59,60]. In some species, different developmental trajectories were found: in chicken nestling alpha diversity increased with age [61,62], while in Bengalese finches (*Lonchura striata domestica*) and American kestrels (*Falco sparverius*), alpha diversity did not vary with nestling age [21,60].

Food treatment did affect beta diversity, but mainly via an interaction with age. When the analyses were limited to the period of food restriction, the direct effect of food on beta diversity was doubled, while the indirect contribution via the interaction with age remained similar. Conforming with other bird studies, age had a strong effect on beta diversity [14,18,21,60,61]. In line with the variation in alpha diversity and the findings in homing pigeons [14], post hoc analyses showed that especially the community compositions of very young nestlings (days 0–2) differed from older nestlings.

### 4.2. Effects of Food Restriction on Taxon Composition Development

Unexpectedly, the relative abundance of the most abundant phyla did not vary with age or food treatment. As is common in birds [63], Firmicutes dominated at all ages, followed by Actinobacteria and Proteobacteria. Relative abundance of Bacteroidetes, also a common phylum in birds [63], was unexpectedly very low (0.3 ± 1.0%). Since especially Bacteroidetes species are adapted to survive periods with limited food resources [26,27], their low abundance suggests that food restriction did not lead to selection for bacteria species with these abilities (contra expectation 1).

Within the 11 most abundant genera, only the relative abundance of *Escherichia-Shigella* and *Clostridium sensu stricto 1* varied with food, being higher under food restriction. That food restriction impacted abundance of *Escherichia-Shigella* was also indicated by the higher abundance of an *Escherichia-Shigella* ASV in young food-restricted nestlings (specificity analyses). Neither *Escherichia-Shigella* nor *Clostridium sensu stricto 1* belong to the Bacteroidetes, and are not known for their abilities to deal with limited resources. *Escherichia-Shigella* species are common inhabitants of vertebrate guts. Some species are beneficial to their hosts, e.g., *E. coli* produces vitamin K [64], but this and other species are mostly known for their pathogenic potential, also within homing pigeons [58]. Although not among the five common pathogens in homing pigeons [58], *Clostridium sensu stricto 1* includes potential pathogenic species. For example, in young pigs, *Clostridium sensu stricto 1* abundance increases under heat stress, which is associated with negative changes in short-chain fatty acid concentrations and blood biochemical indices [65]. The higher abundance of *Escherichia-Shigella* and *Clostridium sensu stricto 1* in food-restricted nestlings suggests that food restriction may result in higher abundance of potential pathogens in the gut microbiome (pro expectation 2).

Relative abundance of four abundant genera varied with age and age^2^: *Bifidobacterium* peaked during the first week of life, while *Corynebacterium 1*, *Actinomyces*, and *Candidatus Arthomitus* peaked after the first week of life. For *Bifidobacterium*, *Actinomyces* and *Candidatus Arthomitus*, the correlation with age was further corroborated, as ASVs of these genera were specific for an age range. All four genera are common members of vertebrate intestines. *Bifidobacterium* is known for its health-promoting abilities: *Bifidobacterium* species produce antimicrobials that may reduce the number of potential pathogens, promote growth and the development of villi in chickens [66,67], and are among the first bacteria that colonize human infant intestines [68]. *Bifodobacteria* are abundant in homing pigeon crop milk (8% [30]), ensuring that young nestlings obtain these beneficial bacteria. *Candidatus Arthomitus* is also a beneficial member of the vertebrate intestine. It belongs to an important group of segmented filamentous bacteria that play a pivotal role in promoting the development of adaptive and innate immune function in murine and poultry species, thereby preventing diseases and promoting growth [69,70]. The increase in *Candidatus Arthomitus* after the first week of life coincides with the decrease in crop milk production, which leads to a reduced influx of parental immunoglobulins [30]. Hence, the increase in *Candidatus Arthomitus* may be beneficial in nestlings by promoting immune function development.

*Lactobacillus*, *Enterococcus*, *Veillonella*, and *Gallibacterium* did not vary with age or food treatment. *Lactobacillus* was the most abundant genus, with high relative abundance at all ages. *Lactobacillus* is by far the largest genus in homing pigeon crop milk (42% [30]), which may explain why *Lactobacillus* abundance was high throughout development. *Lactobacillus* species are very beneficial to development and associated with higher body mass, better health, and longer survival in young birds [71,72]. *Enterococcus*, *Veillonella*, and *Gallibacterium* also occur in relatively high abundance in homing pigeon crop milk (6–9% [30]). *Enterococcus* is a common inhabitant of vertebrate guts, and although known for its pathogenic potential [58], some *Enterococcus* strains have beneficial functional potentials for their host, including disease prevention [73,74]. *Veillonella*, another beneficial commensal of vertebrate guts, promotes homeostasis by producing short-chain fatty acids via lactate fermentation [75]. Lastly, *Gallibacterium* is a normal inhabitant of the cloaca in poultry. A specific species, *G. anatis*, is known to become pathogenic in poultry when the host suffers immunosuppression, is co-infected, or experiences stress [76]. However, in rock pigeon nestlings, *G. genomosp. 3* was the most abundant *Gallibacterium* ASV, and *G. anatis* was not detected.

### 4.3. Potential Implications for Avian Ecological Studies

It is becoming increasingly clear that host microbiomes, such as the gut microbiome, play a role in the ecology and evolution of natural populations of birds. During development, the gut microbiome is one of the factors that determine the physiological and behavioral phenotype of offspring and thereby may impact their fitness [77]. For example, great tit nestlings that experimentally received the beneficial, host-derived gut microbe *Lactobacillus kimchicus* had a more diverse gut microbiome and were heavier than control nestlings, which was expected to increase their chance of survival [78]. This indicates that there is a link between the gut microbiome and nestling body mass, and thus with fitness. It is therefore important that we gain more insight into the development of the gut microbiome and the impact of environmental factors thereupon. Our study contributes to this, and shows that periods of food limitation early in life do impact the development of the gut microbiome, but only during the period of food limitation. We expect that also in natural bird populations the effects of food limitation on gut microbiome development may be resolved once the food resources are restored. However, when food is severely limited, it is likely that the offspring’s diet composition also changes considerably. This combination may have a larger and/or potentially longer-term impact on the gut microbiome development than shown here.

## 5. Conclusions

We investigated the impact of food restriction during the first week of life on the development of the gut microbiome in rock pigeon nestlings. We expected that food restriction would either increase the competition among microbes, resulting in lower diversity (1), or suppress the immune system, resulting in higher diversity, accompanied potentially by an increase in pathogenic bacteria (2). We expected that after ending the food restriction, differences in the gut microbiome would diminish rapidly (3) or be maintained if initial colonization differed between the food treatments and impacted gut microbiome development (4). Food restriction affected gut microbiome development during the first week of life: the difference in alpha diversity between nestlings and adults was higher, with higher diversity in nestlings, and two potential pathogens were more abundant in the first week of life. These effects are potentially linked to immune function suppression (expectation 2), but note that we did not determine immune function indices of these nestlings. Bacterial community composition was also affected by food treatment, both directly and via an interaction with age, whereby especially very young nestlings (0 and 2 days) differed from older nestlings. However, the major factor influencing gut microbiome development was age, with significant changes occurring during the first week of life, aligning with developmental trajectories in alpha and beta diversity observed in other bird species. Most of the very abundant genera did not vary with food treatment and were beneficial for health and immune function development. The differences in alpha and beta diversity disappeared when food restriction was ended (expectation 3), indicating a large impact of diet quality and quantity on gut microbiome development.

In conclusion, food restriction had significant and long-term effects on nestling body mass in rock pigeons and a short-term effect on the gut microbiome, which diminished when food restriction was ended. Our findings contribute valuable insights into the dynamic relationship between developmental conditions and the avian gut microbiome.

## Figures and Tables

**Table 1:** LMM analysis of the relationships between alpha diversity and age, age^2^, food treatment, testosterone treatment, standardized body mass, and the interaction terms age*food and food*testosterone in rock pigeon nestlings.

Alpha Diversity	Predictors, Final Model ^1^	*df*	*F*	*p*	Marginal *R*^2^	Conditional *R*^2^
Richness	Age Age^2^	1, 86 1, 86	10.44 7.04	0.002 0.010	0.114	0.157
Shannon	Age Age^2^	1, 86 1, 86	7.02 4.41	0.010 0.039	0.090	0.090
Chao1	Age Age^2^	1, 86 1, 86	7.54 4.93	0.007 0.029	0.089	0.123
Faith’s PD	Age Age^2^	1, 86 1, 86	9.81 6.57	0.002 0.012	0.106	0.173

^1^ Presented are the fixed factors and marginal and conditional *R*^2^ of the final models. Individual bird ID was included as random factor. Units of the presented predictors: age, days.

**Table 2:** PERMANOVA analyses of the relationships between beta-diversity indices of nestlings and adults, and age and food treatment.

Beta Diversity Indices	Predictors, Final Model ^1^	*R* ^2^	*F*	*p*
Jaccard	Age Food Age*Food	0.197 0.016 0.088	2.78 2.01 1.25	0.001 0.003 0.012
Bray–Curtis	Age Food Age*Food	0.256 0.019 0.087	3.97 2.58 1.34	0.001 0.002 0.015
Unweighted UniFrac	Age Food	0.195 0.015	2.69 1.88	0.0001 0.016
Weighted UniFrac	Food	0.029	3.19	0.004

^1^ Only the fixed factors of the final models are presented. Units of the predictors: age was a categorical variable, days for nestlings plus adult; food treatment, normal or restricted.

**Table 3:** LMM analysis of the relationships between nestlings’ genus proportions and age, age^2^, food and testosterone treatment, standardized body mass, and the interaction terms age*food and food*testosterone.

Genus	Predictors, Final Model ^1^	*df*	*F*	*p*	Marginal *R*^2^	Conditional *R*^2^
*Actinomyces*	Age Age^2^	1, 86 1, 86	10.54 8.89	0.002 0.004	0.164	0.164
*Bifidobacterium*	Age	1, 87	6.56	0.012	0.062	0.062
*Corynebacterium 1*	Age Age^2^	1, 86 1, 86	11.20 5.81	0.001 0.018	0.147	0.147
*Candidatus Arthomitus*	Age Age^2^	1, 86 1, 86	43.02 24.28	<0.0001 <0.0001	0.380	0.444
*Clostridium sensu stricto 1*	Age Food Age*Food	1, 86 1, 10 1, 86	2.14 1.34 11.45	0.147 0.275 0.001	0.127	0.243
*Escherichia-Shigella*	Food	1, 4	7.78	0.049	0.172	0.260

^1^ Fixed factors and marginal and conditional *R*^2^ of the final models with significant factors are presented. Individual bird ID was included as random factor. Units of the predictors: age, days; food treatment, normal or restricted.

## Data Availability

The original data presented in the study are openly available on the DataverseNL database via the public-accessible persistent link https://doi.org/10.34894/EMBJKR. This are the following data and information: the raw sequence datasets (Fasta files) generated and/or analyzed in the current study; the QIIME2 and the R scripts; the ASV community table file for the creation of a *phyloseq* object (json format BIOM file); the phylogenetic tree file for analysis in R; the metadata for the creation of a *phyloseq* object; data files with nestling traits and alpha diversity; and an Excel file with the key to the column names of the metadata and data files.

## Supplementary Materials

The following supporting information can be downloaded at https://www.mdpi.com/article/10.3390/microorganisms13061191/s1. Supplementary Materials and Methods; Figure S1: Rarefaction curves for richness (A) and Shannon index (B); Figure S2: The effects of food restriction on the development of Chao1 and Faith’s phylogenetic diversity; Figure S3: The variation in Jaccard similarities (A) and unweighted UniFrac distances (B) with age and food treatment in nestlings and adults; Figure S4: Visualization of post hoc analyses of variation in Jaccard similarities (A), Bray–Curtis dissimilarities (B), and unweighted UniFrac distances (C) with age in nestlings and adults; Figure S5: The relative abundance of phyla under normal food (A) and food restriction (B) conditions; Figure S6: Distribution of proportions with age of ASVs specific for nestling age range within nestlings under normal food conditions (Table S6); Figure S7: Distribution of proportions with age for ASVs specific for nestling age range within nestlings under food-restriction conditions (Table S6); Figure S8: Distribution of proportions of the Streptococcus genus with age; Table S1: Mean body mass (g) and tarsus length (mm) of rock pigeon nestlings per age and food treatment group; Table S2: LMM analysis of relationships between nestling parameters (body mass and tarsus length), and age, food regime, and testosterone treatment; Table S3: LMM analysis of relationships between differences in alpha diversity between rock pigeon nestlings and adults (CH-AD), and age, food and testosterone treatment; Table S4: Results of PERMANOVA analyses of relationships between beta-diversity indices of day 0–7 old nestlings and age and food treatment; Table S5: Proportions and prevalence of the most abundant genera in nestlings and adults; Table S6: Overview of ASVs specific for a limited age range per food treatment. References [13,33,39,40,79,80] are cited in the Supplementary Materials.

## Abbreviations

The following abbreviation is used in this manuscript:

GM	gut microbiome
