# Supplementary material for: Gut Microbiome Development in Rock Pigeons: Effects of Food Restriction Early in Life
Source: Microorganisms. 2025 May 23;13(6):1191. doi: 10.3390/microorganisms13061191 (PMC12194888; doi:10.3390/microorganisms13061191)
Supplement: Supplementary file 1 [file microorganisms-13-01191-s001.zip › Figure S2.pdf]

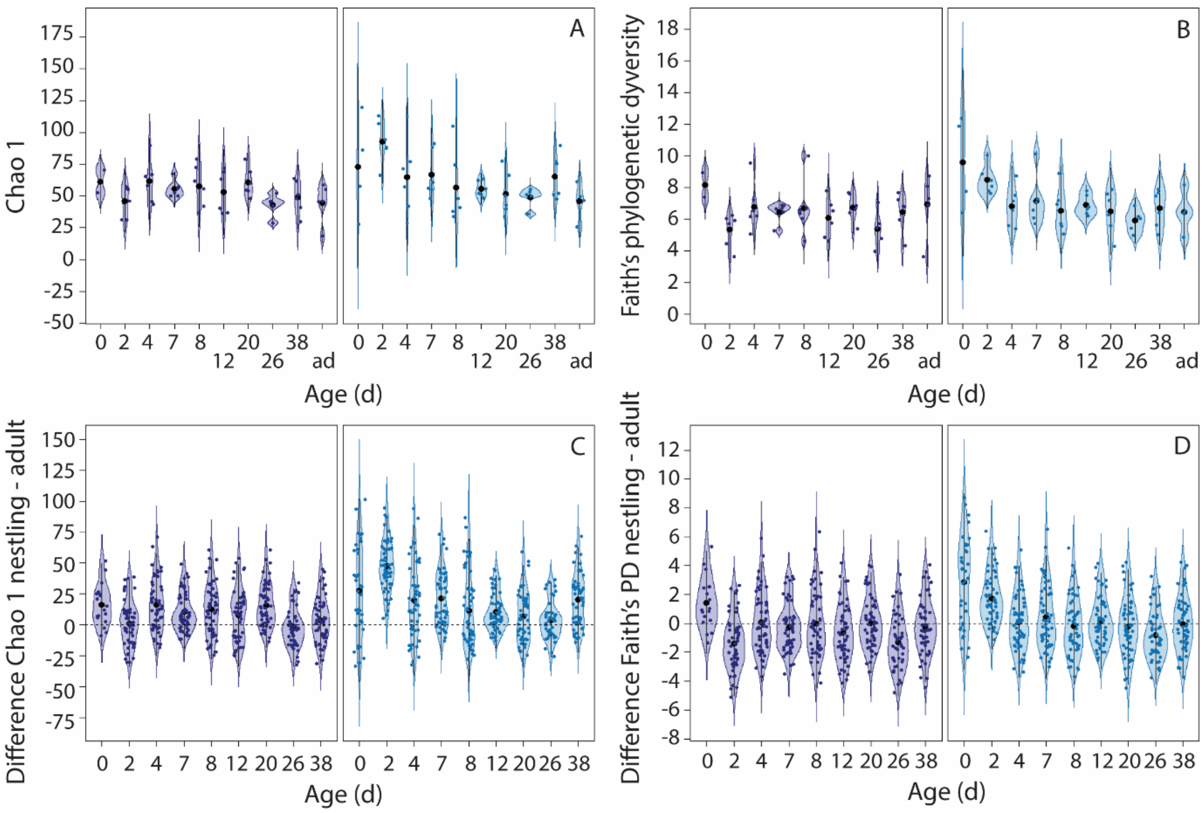

Figure S2: The effects of food restriction on the development of Chao 1 and Faith's phylogenetic diversity. Presented are the variation in Chao1 index (A) and Faith's phylogenetic diversity (B) with age, and the variation in the difference in Chao1 (C) and Faith's phylogenetic diversity (D) between nestlings and adults with age. Left panels, normal food treatment (dark blue violins), right panels, food restriction treatment (light blue violins). Black symbols indicate the mean, the blue symbols are the raw data. The dashed line in the bottom panels indicates no difference between nestlings and adults. Note that the adults are included in panels A and B but not in the LMMs, where age is included as continuous variable. Sample sizes panels A and B: per age per treatment group 6 chicks (3 nests), except for day 0 (2 normal food chicks and 3 food restricted chicks), and day 26 (5 chicks per age per treatment group). Sample sizes panels C and D: for each chick presented in A and B, the differences in alpha diversity with 9 adults are given, thus 6*9 = 54 data points per age and treatment group, except for day 0 (18 data points for normal food chicks, and 27 data points for food restricted chicks) and day 26 (45 data points per age per treatment group).
