# Supplementary material for: Gut Microbiome Development in Rock Pigeons: Effects of Food Restriction Early in Life
Source: Microorganisms. 2025 May 23;13(6):1191. doi: 10.3390/microorganisms13061191 (PMC12194888; doi:10.3390/microorganisms13061191)
Supplement: Supplementary file 1 [file microorganisms-13-01191-s001.zip › Figure S3.pdf]

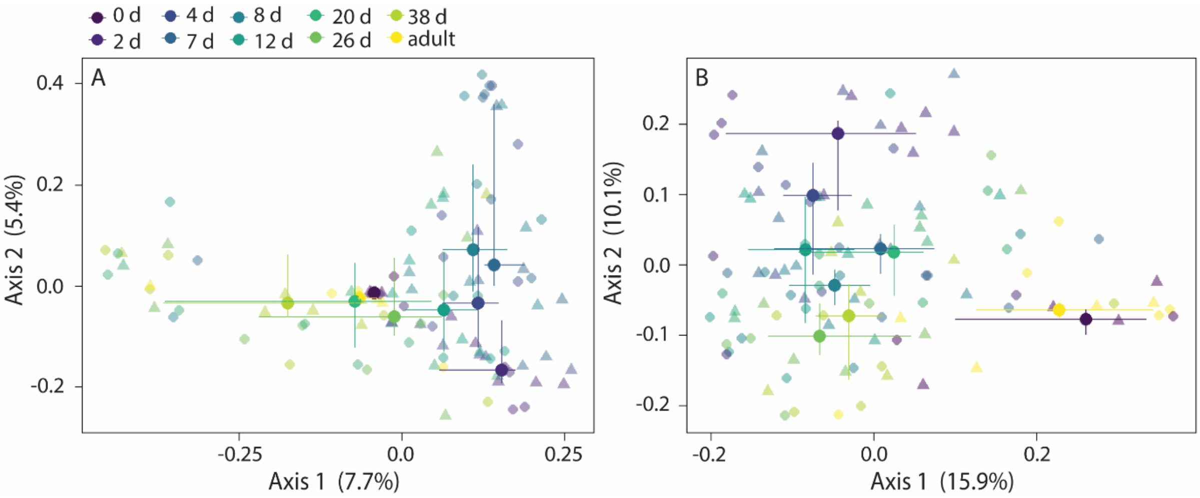

Figure S3: The variation in Jaccard similarities (A) and unweighted UniFrac distances (B) with age and food treatment in nestlings and adults. Large symbols present medians, the error bars the 25% and 75% quantiles. Transparent symbols present the underlying data. Symbols: circles, normal food treatment; triangles, food restriction treatment. Sample sizes: per age per treatment group 6 chicks (3 nests), except for day 0 (2 normal food chicks and 3 food restricted chicks), and day 26 (5 chicks per age per treatment group); and 9 adults.
