# Supplementary material for: Gut Microbiome Development in Rock Pigeons: Effects of Food Restriction Early in Life
Source: Microorganisms. 2025 May 23;13(6):1191. doi: 10.3390/microorganisms13061191 (PMC12194888; doi:10.3390/microorganisms13061191)
Supplement: Supplementary file 1 [file microorganisms-13-01191-s001.zip › Figure S4.pdf]

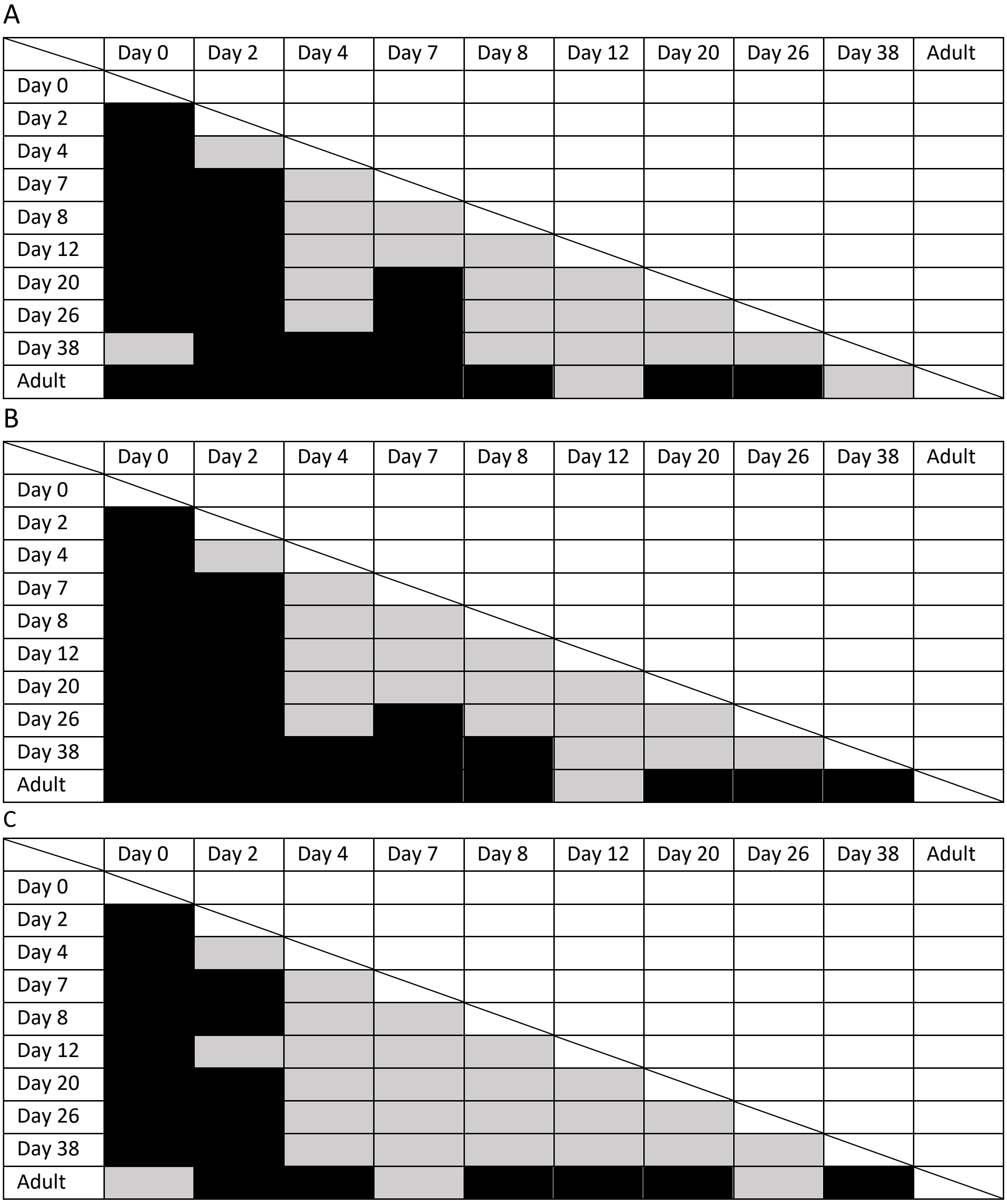

Figure S4: Visualization of the posthoc analyses of the variation in Jaccard similarities (A), Bray-Curtis dissimilarities (B), and unweighted UniFrac distances (C) with age in nestlings and adults. Black blocks indicate a significant difference between two ages, while grey blocks indicate no difference between two ages.
