# Supplementary material for: Gut Microbiome Development in Rock Pigeons: Effects of Food Restriction Early in Life
Source: Microorganisms. 2025 May 23;13(6):1191. doi: 10.3390/microorganisms13061191 (PMC12194888; doi:10.3390/microorganisms13061191)
Supplement: Supplementary file 1 [file microorganisms-13-01191-s001.zip › Figure S5.pdf]

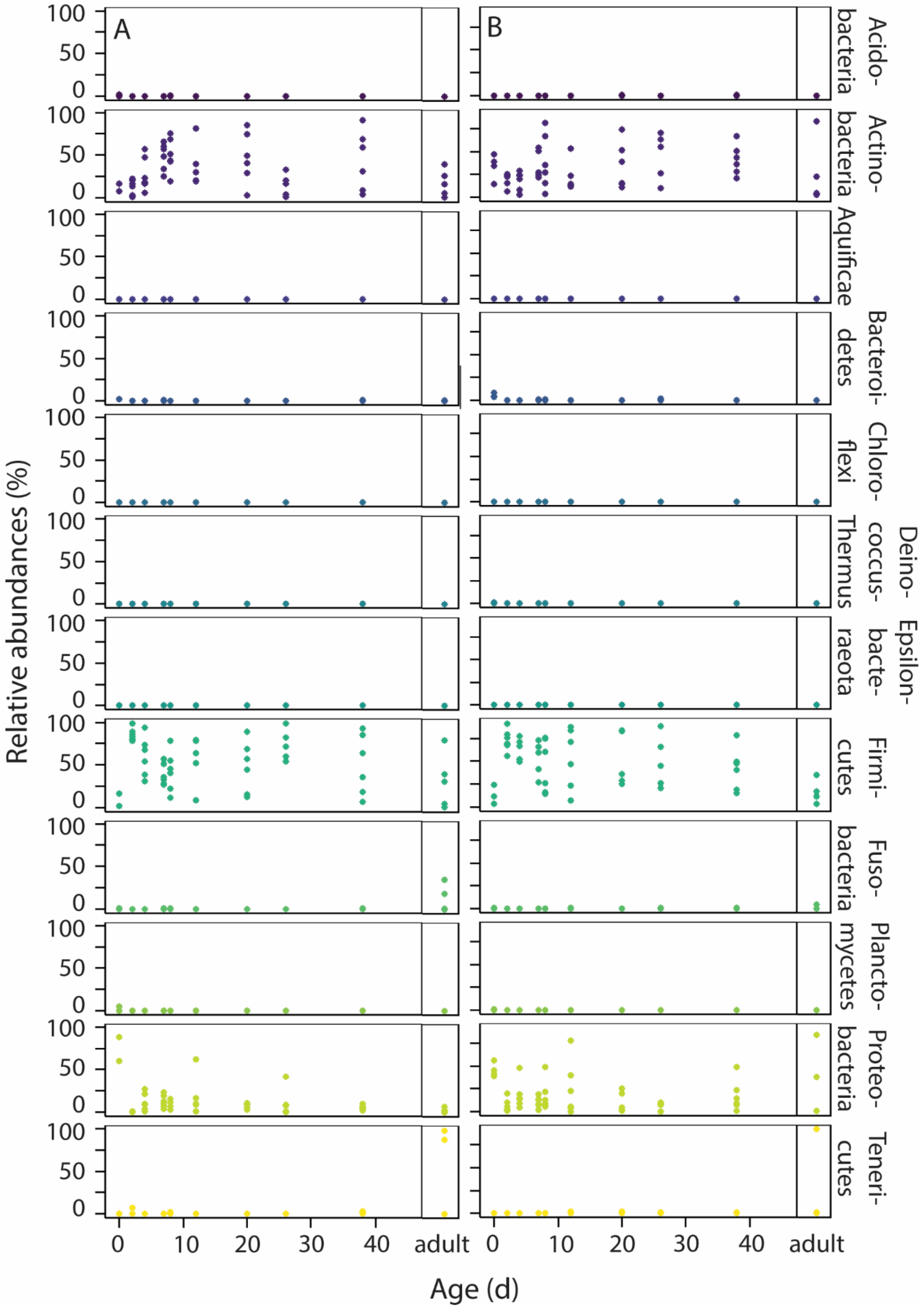

Figure S5: The relative abundances of the phyla under normal food (A) and food restriction (B) conditions. Sample sizes: per age per treatment group 6 chicks (3 nests), except for day 0 (2 normal food chicks and 3 food restricted chicks), and day 26 (5 chicks per age per treatment group); and 9 adults.
