# Supplementary material for: Gut Microbiome Development in Rock Pigeons: Effects of Food Restriction Early in Life
Source: Microorganisms. 2025 May 23;13(6):1191. doi: 10.3390/microorganisms13061191 (PMC12194888; doi:10.3390/microorganisms13061191)
Supplement: Supplementary file 1 [file microorganisms-13-01191-s001.zip › Figure S7.pdf]

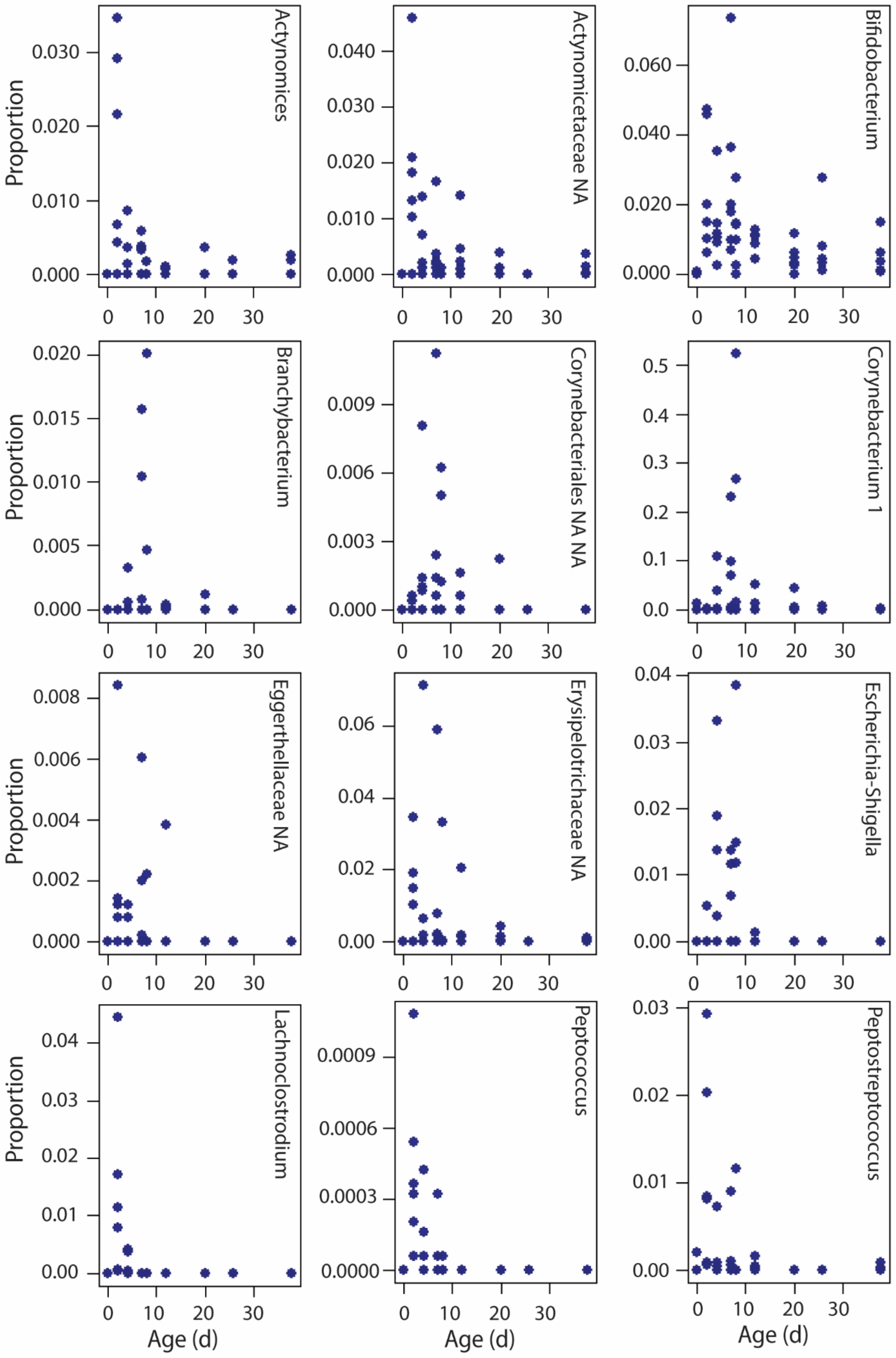


**Figure S7: Distribution of proportions with age for the ASVs specific for a nestling age range within nestlings under food restriction conditions (Table S6).** The ASVs are presented combined per genus (genera names given in the panels) for visualization purposes. Sample sizes: per age per treatment group 6 chicks (3 nests), except for day 0 (2 normal food chicks and 3 food restricted chicks), and day 26 (5 chicks per age per treatment group).
