# Supplementary material for: Gut Microbiome Development in Rock Pigeons: Effects of Food Restriction Early in Life
Source: Microorganisms. 2025 May 23;13(6):1191. doi: 10.3390/microorganisms13061191 (PMC12194888; doi:10.3390/microorganisms13061191)
Supplement: Supplementary file 1 [file microorganisms-13-01191-s001.zip › Supplementary Materials and Methods - proofs.pdf]

Supplementary Materials and Methods

Egg injections

In half of the experimental eggs (T- eggs), we increased the yolk testosterone levels to levels comparable with those naturally present in the second-laid eggs following the procedure described by Hsu, Dijkstra & Groothuis (2016). Briefly, eggs were placed on their side for a few minutes to allow the yolk to float up. The eggshell was sterilized at the blunt end with 70% alcohol and punctured with the sterile needle of a 0.5ml U-100 insulin syringe (0.33mm needles×12.7mm syringe, BD micron-Fine™). We injected 50µl sterile testosterone solution (crystalline testosterone [Fluka] dissolved in 50ml sterile sesame oil at a concentration of 920ng testosterone/ml oil) in the yolk of T-eggs, and 50µl pure sterile sesame oil in the yolk of control eggs (C-eggs). After withdrawing the syringe, the hole in the eggshell was sealed with a small piece (~25 mm^2) of artificial skin (Hansaplast ™). The hatching success of treated eggs was similar for both groups across three experiments (2011-2013; on average 53.85±1.96% for T-eggs and 48.85 ± 2.83% for C-eggs; $t_{3,562}$=2.510, *P*=0.074; [33], which is close to the natural hatching success in feral pigeon (*Columbia livia*; 68.5%; [79].

DNA isolation and 16s rRNA gene amplicon sequencing

We randomized the samples before DNA extraction. DNA was isolated from the feces and cloacal swabs using the PowerSoil® DNA kit (MoBio, Carlsbad, CA, USA) according to the manufacturer's instruction with the exception that we added extra zirconia/silica beads (~0.25g; Ø 0.1 mm; Thistle scientific, Warwickshire, UK) to the first reaction tube, and that the sample was bead beaten three times one minute instead of three minutes continuously to prevent the sample from heating up too much. We added the feces plus the fiber material of the swab and all soluble contents of the vial to the first reaction tube. For cloacal swabs the fiber material and all soluble contents were added. The extracted DNA was stored at -20°C until further use.

We quantified sample DNA concentrations using the Quant-it PicoGreen dsDNA kit (Molecular Probes, Invitrogen, Eugene, OR, USA) and normalized the DNA concentrations in the subsequent PCR to 1ng template DNA per 25µl reaction. The samples were randomized again before amplifying the V4/V5 region of the 16S rRNA gene in triplicate using the primers 515F and 926R [39,40] with Illumina adaptors at the 5′-end. We used the following thermal cycling protocol: 5 min at 95°C, 35 cycles with 40 s at 95°C, 45 s at 56°C, 40 s at 72°C, followed by 10 min at 72°C. DNA concentrations were very low in the first feces samples [13]. Therefore, we added 10µl template of the first feces samples and 2µl template of the cloacal swab samples to the 5µl PCR mix, and added for the latter also 8µl nuclease-free water to reach a total PCR volume of 15µl. The triplicates were pooled after the PCR, and sent after purification (QIAquick gel extraction Kit, QIAGEN GmbH, Hilden, Germany) to GenoToul (INRA, Toulouse, France) for library preparations and Illumina sequencing

using 2×250bp v2 chemistry (114 pigeon samples, a negative control swab and 4 negative PCR controls; 5 pigeon samples contained too little DNA to be sequenced). At GenoToul, the sequence reads were demultiplexed and quality filtered using the default settings in QIIME.

Sequence data checks

We explored the data by inspecting rare ASVs as based on number of read counts and ASV prevalence. Because we used DADA2, the initial data contained no singletons. After removing the contaminant ASVs and the negative control samples, there were 10 singleton ASVs (0.6%) and 197 doubleton ASVs (11.9%), indicating that few ASVs had low read counts. However, despite having nine samples per chick, many ASVs had a very low prevalence as 77.7% of the ASVs occurred in only one sample. Since we have for the nestlings multiple samples per individual, this suggests that there was a high variability between the samples both within and among individuals. The low prevalence percentage is comparable to percentages previously found by us, such as in captive homing pigeons (4 samples per individual, 81.3%; [13] and adult free-living feral pigeons (61.3%; Dietz pers. comm.), and comparable to those found in a variety of other species [80]. After rarefying the percentage of singletons was 30.7%, while 14.5% of the ASVs were doubletons. The percentage of very low prevalence ASVs was comparable to before rarefying, with 70.8% of the ASVs occurring in one sample.
