# Supplementary material for: Gut Microbiome Development in Rock Pigeons: Effects of Food Restriction Early in Life
Source: Microorganisms. 2025 May 23;13(6):1191. doi: 10.3390/microorganisms13061191 (PMC12194888; doi:10.3390/microorganisms13061191)
Supplement: Supplementary file 1 [file microorganisms-13-01191-s001.zip › Table S1.pdf]

Table S1. Mean body mass (g) and tarsus length (mm) of rock pigeon nestlings per age and food treatment group.

Age	Food treatment	Body mass	Tarsus length	N
0	Normal	11.3 ± 1.2	11.7 ± 0.5	6
0	Restricted	11.3 ± 1.6	11.3 ± 0.5	6
2	Normal	23.0 ± 2.1	14.2 ± 0.8	6
2	Restricted	24.3 ± 2.7	14.7 ± 0.8	6
4	Normal	47.3 ± 5.2	18.0 ± 0.6	6
4	Restricted	48.0 ± 6.0	18.7 ± 0.8	6
8	Normal	90.5 ± 7.9	24.8 ± 0.8	6
8	Restricted	69.5 ± 12.7	24.2 ± 1.2	6
12	Normal	137.3 ± 13.0	29.8 ± 1.0	6
12	Restricted	104.0 ± 18.8	28.5 ± 1.4	6
20	Normal	205.3 ± 11.9	34.8 ± 0.8	6
20	Restricted	153.0 ± 24.1	32.7 ± 1.4	6
26	Normal	240.7 ± 9.3	35.7 ± 0.8	6
26	Restricted	184.2 ± 26.2	34.2 ± 1.2	6
38	Normal	242.2 ± 9.8	34.8 ± 0.8	6
38	Restricted	197.2 ± 19.7	34.0 ± 1.4	6
