# Supplementary material for: Gut Microbiome Development in Rock Pigeons: Effects of Food Restriction Early in Life
Source: Microorganisms. 2025 May 23;13(6):1191. doi: 10.3390/microorganisms13061191 (PMC12194888; doi:10.3390/microorganisms13061191)
Supplement: Supplementary file 1 [file microorganisms-13-01191-s001.zip › Table S2.pdf]

Table S2. LMM analysis of the relationships between nestling parameters (body mass and tarsus length), and age, food regime, and testosterone treatment.

**Nestling parameters**	**Predictors final model[1]**	**Df**	***F***	***P***	***Marginal R^2***	***Conditional R^2***
**Body mass**	Age	1,81	974.42	<0.0001	0.966	0.972
	Age^2	1,81	246.70	<0.0001		
	Food	1,10	0.54	0.48		
	Age*Food	1,81	49.43	<0.0001		
**Tarsus**	Age	1,81	2700.04	<0.0001	0.980	0.984
	Age^2	1,81	1242.17	<0.0001		
	Food	1,10	0.05	0.83		
	Age*Food	1,81	5.64	0.02		

[1]The initial model contained age, food and testosterone treatment, the interaction terms age*food treatment and testosterone treatment*food treatment (fixed factors), and individual nestling nested within aviary (random factors). Aviary never contributed significantly to the variation. We present the fixed factors, and marginal and conditional R^2 of the final models. Units of the presented predictors: age, days; body mass, g; food treatment, normal or restricted food.
