# Supplementary material for: Gut Microbiome Development in Rock Pigeons: Effects of Food Restriction Early in Life
Source: Microorganisms. 2025 May 23;13(6):1191. doi: 10.3390/microorganisms13061191 (PMC12194888; doi:10.3390/microorganisms13061191)
Supplement: Supplementary file 1 [file microorganisms-13-01191-s001.zip › Table S3.pdf]

Table S3. LMM analysis of the relationships between the differences in alpha diversities between rock pigeon nestlings and adults (CH-AD), and age, food and testosterone treatment.

CH-AD in alpha diversity	Predictors final model[1]	Df	*F*	*P*	*Marginal* R^2	*Conditional* R^2
**Richness**	Age	1,886	4.99	0.026	0.066	0.120
	Food	1,10	9.41	0.012		
	Food*Age	1,886	5.74	0.017		
**Shannon**	Age	1,887	9.99	0.002	0.011	0.023
**Chao1**	Age	1,886	4.90	0.027	0.075	0.119
	Food	1,10	12.75	0.005		
	Food*Age	1,886	4.92	0.027		
**Faith's PD**	Age	1,86	0.62	0.431	0.011	0.023
	Food	1,10	14.94	0.003		
	Food*Age	1,86	13.49	0.003		

[1]The initial model contained age, age^2, food and testosterone treatments, standardized body mass, the interaction terms age*food treatment and testosterone treatment*food treatment (fixed factors), and individual bird nested within aviary (random factors). Aviary contributed never significantly to the variation. We present the fixed factors, and marginal and conditional R^2 of the final models. Units of the predictors: age, days; food treatment, normal or restricted.
