# Supplementary material for: Gut Microbiome Development in Rock Pigeons: Effects of Food Restriction Early in Life
Source: Microorganisms. 2025 May 23;13(6):1191. doi: 10.3390/microorganisms13061191 (PMC12194888; doi:10.3390/microorganisms13061191)
Supplement: Supplementary file 1 [file microorganisms-13-01191-s001.zip › Table S4.pdf]

Table S4. Results of the PERMANOVA analyses of the relationships between the beta diversity indices of day 0-7 old nestlings, and age and food treatment.

Beta diversity indices	Predictors final model[1]	R^2	*F*	*P*
**Jaccard**	Age	0.144	2.24	<0.001
	Food	0.045	2.10	<0.001
	Age*Food	0.082	1.28	0.017
**Bray-Curtis**	Age	0.268	5.25	<0.001
	Food	0.066	3.85	<0.001
	Age*Food	0.087	1.71	0.009
**Unweighted UniFrac**	Age	0.194	3.14	<0.001
	Food	0.045	2.18	<0.001
**Weighted UniFrac**	Age	0.522	16.27	<0.001
	Food	0.040	3.76	0.006
	Age*Food	0.075	2.33	0.012

[1]We present the fixed factors of the final models; the initial models contained age, food treatment and their interaction term age*food treatment. Units of the presented predictors: age was entered as a categorical variable, days; food treatment, normal or restricted. Individual was not significant for unweighted and weighted UniFrac, and thus standard PERMANOVA analyses were done.
