# Supplementary material for: Gut Microbiome Development in Rock Pigeons: Effects of Food Restriction Early in Life
Source: Microorganisms. 2025 May 23;13(6):1191. doi: 10.3390/microorganisms13061191 (PMC12194888; doi:10.3390/microorganisms13061191)
Supplement: Supplementary file 1 [file microorganisms-13-01191-s001.zip › Table S5.pdf]

Table S5. Proportions and prevalence of the most abundant genera in nestlings and adults.

Genus number	Phylum	Class	Order	Family	Genus	Mean proportion[1]	Prevalence
e80fc06fb3fbcdc644bd8379fbcae78b	*Actinobacteria*	*Actinobacteria*	*Actinomycetales*	*Actinomycetaceae*	*Actinomyces*	0.038 ± 0.055	107
5e29d9c0d3e74e4fa04e7878dc190536			*Bifidobacteriales*	*Bifidobacteriaceae*	*Bifidobacterium*	0.023 ± 0.030	102
d7141f31a41d78e1bea19258d3193f50			*Corynebacteriales*	*Corynebactericeae*	*Corynebacterium 1*	0.216 ± 0.215	109
6f2f05b4bea9a2c9f8172233d4ad89a7	*Firmicutes*	*Bacilli*	*Lactobacillales*	*Entercoccaceae*	*Enterococcus*	0.097 ± 0.130	108
994fe0a583b7fd1b01e8c2d3d3e33ef8				*Lactaobacillalceae*	*Lactobacillus*	0.244 ± 0.245	108
2cc06a527bdde8c8688917fd44b76a9f		*Clostridia*	*Clostridiales*	*Clostridiaceae 1*	*Candidatus Arthomitus*	0.054 ± 0.140	63
1260d3947c4a612fa25b1ee09bba6e89					*Clostridium sensu stricto 1*	0.024 ± 0.063	85
bd18131f0bbcfe89abb6242c10d17273		*Negativicutes*	*Selenomonadales*	*Veillonellaceae*	*Veillonella*	0.037 ± 0.042	108
7bae1eeda819cc117db4bcc085bddd01	*Proteobacteria*	*Gammaproteobacteria*	*Enterobacteriales*	*Enterobactericeae*	*Escherichia-Shigella*	0.078 ± 0.158	103
b007bbd118550b97c1f7aba24126b456			*Pasteurellales*	*Pasteurellaceae*	*Gallibacterium*	0.031 ± 0.050	102
b9e215918d5f7e7dd4cfc23eb2ade886	*Tenericutes*	*Mollicutes*	*Mysoplasmatales*	*Mycoplasmataceae*	*Candidatus Bacilloplasma*	0.028 ± 0.153	55

[1]The mean proportion is presented ± standard deviation.
