# Supplementary material for: Gut Microbiome Development in Rock Pigeons: Effects of Food Restriction Early in Life
Source: Microorganisms. 2025 May 23;13(6):1191. doi: 10.3390/microorganisms13061191 (PMC12194888; doi:10.3390/microorganisms13061191)
Supplement: Supplementary file 1 [file microorganisms-13-01191-s001.zip › Table S6.pdf]

Table S6. Overview of the ASVs specific for a limited age range per food treatment.

ASV number	Phylum	Class	Order	Family	Genus[1]	*P*	Mean proportion[2]	Peak[3]
Normal food treatment								
18137dccfdcbb20b71b9f4b003b1a0bc	*Actinobacteria*	*Actinobacteria*	*Actinomycetales*	*Actinomycetaceae*	*Actinomyces*	0.019	0.0003 ± 0.0008	2-12
bd2d15c1a872fb07c72d67292c4f971a					*Actinomyces*	0.017	0.0035 ± 0.0070	2-12
67f4dd5e58c3b83376bbea4a83a1bd62					*NA*	0.027	0.0021 ± 0.0047	4-12
20c88d4520e25c793987f54df9d7a5d1			*Bifidobacteriales*	*Bifidobacteriaceae*	*Bifidobacterium*	0.019	0.0021 ± 0.0054	2-12
5e29d9c0d3e74e4fa04e7878dc190536					*Bifidobacterium*	0.017	0.0191 ± 0.0294	2-12
82e8cf69da6ecb77fae20a04f3b63f6e			*Corynebacteriales*	*Corynebacteriaceae*	*Corynebacterium 1*	0.017	0.1070 ± 0.1684	4-8
2ba912e269f158f68372fc0f96777e70				NA	NA	0.019	0.0033 ± 0.0119	4-8
6e326a6b53a4d0e352bb43a02f389e1a	*Firmicutes*	*Bacilli*	*Bacillales*	*Staphylococcaceae*	*Staphylococcus*	0.017	0.0030 ± 0.0039	4-20
55466caa16236bbd6ca2c65ea96f5bb4		*Clostridia*	*Clostridiales*	*Lachnospiraceae*	NA	0.022	0.0009 ± 0.0034	2-8
7d0e16101d066fc43d3c5faf52046590	*Proteobacteria*	*Gammaproteobacteria*	*Pasteurellales*	*Pasteurellaceae*	*Gallibacterium*	0.022	0.0018 ± 0.0044	4-12
Food restriction treatment								
bd2d15c1a872fb07c72d67292c4f971a	*Actinobacteria*	*Actinobacteria*	*Actinomycetales*	*Actinomycetaceae*	*Actinomyces*	0.025	0.0027 ± 0.0069	2-4
67f4dd5e58c3b83376bbea4a83a1bd62					*NA*	0.034	0.0038 ± 0.0080	2-12
5e29d9c0d3e74e4fa04e7878dc190536			*Bifidobacteriales*	*Bifidobacteriaceae*	*Bifidobacterium*	0.025	0.0120 ± 0.0143	2-8
a93e17714b1ad13879640acae86f21e3			*Corynebacteriales*	*Corynebacteriaceae*	*Corynebacterium 1*	0.013	0.0304 ± 0.0883	4-7
2ba912e269f158f68372fc0f96777e70				NA	NA	0.013	0.0009 ± 0.0022	4-7
629a3a6a93c38e9ffb54f10ab2e67283			*Micrococcales*	*Dermabacteraceae*	*Brachybacterium*	0.013	0.0011 ± 0.0038	7-8
8da6e021e5f19e75270493013b831824		*Coriobacteriia*	*Coriobacteriales*	*Eggerthellaceae*	NA	0.038	0.0006 ± 0.0016	2-12
9e0aa79a34a0c11a6a5317f4a1751431	*Firmicutes*	*Clostridia*	*Clostridiales*	*Lachnnospiraceae*	*Lachnoclostridium*	0.013	0.0018 ± 0.0068	2-4
57b31ef718d0ded98e31a112237d4038				*Peptococcaceae*	*Peptococcus*	0.013	0.0007 ± 0.0019	2-7
6549de680de9ee33e5702a623d2e2a35					*Peptostreptococcus*	0.022	0.0020 ± 0.0054	2-8
ad30128700b980cccc625cf374d048eb		*Erysipelotrichia*	*Erysipelotrichales*	*Erysipelotrichaceae*	NA	0.030	0.0058 ± 0.0145	2-12
e0acacf32cd361ae1f5ccff5ba1ad710	*Proteobacteria*	*Gammaproteobacteria*	*Enterobacteriales*	*Enterobacteriaceae*	*Escherichia-Shigella*	0.013	0.0034 ± 0.0081	2-8

[1]The SILVA data base provides few species names and therefore taxa information is given to genus level. [2]The mean proportion is given ± standard deviation. [3]Peak is the age range during which the mean proportion for that genus was the highest (thus ASVs combined), which was visually deducted from plots of ASV proportion versus age (Figs. S6, and S7). Sample size was 49 for nestlings under normal food treatment and 50 for nestlings under food restriction.
