# Supplementary figures and images for: Gut Microbiome Development in Rock Pigeons: Effects of Food Restriction Early in Life
Source: Microorganisms. 2025 May 23;13(6):1191. doi: 10.3390/microorganisms13061191 (PMC12194888; doi:10.3390/microorganisms13061191)

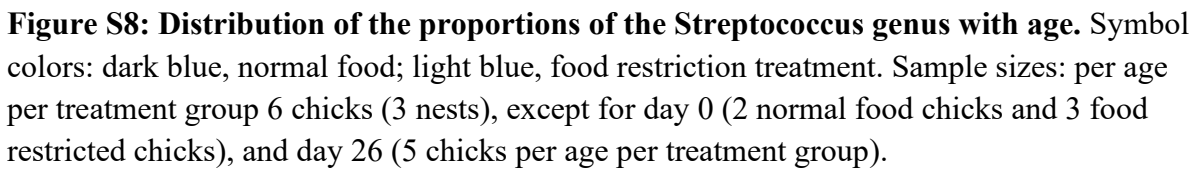

Supplement: Supplementary file 1 [file microorganisms-13-01191-s001.zip › Figure S8.pdf]
