# Supplementary material for: Gut Microbiome Development in Rock Pigeons: Effects of Food Restriction Early in Life
Source: Microorganisms. 2025 May 23;13(6):1191. doi: 10.3390/microorganisms13061191 (PMC12194888; doi:10.3390/microorganisms13061191)
Supplement: Supplementary file 1 [file Figure S1.pdf › Figure S1.pdf]

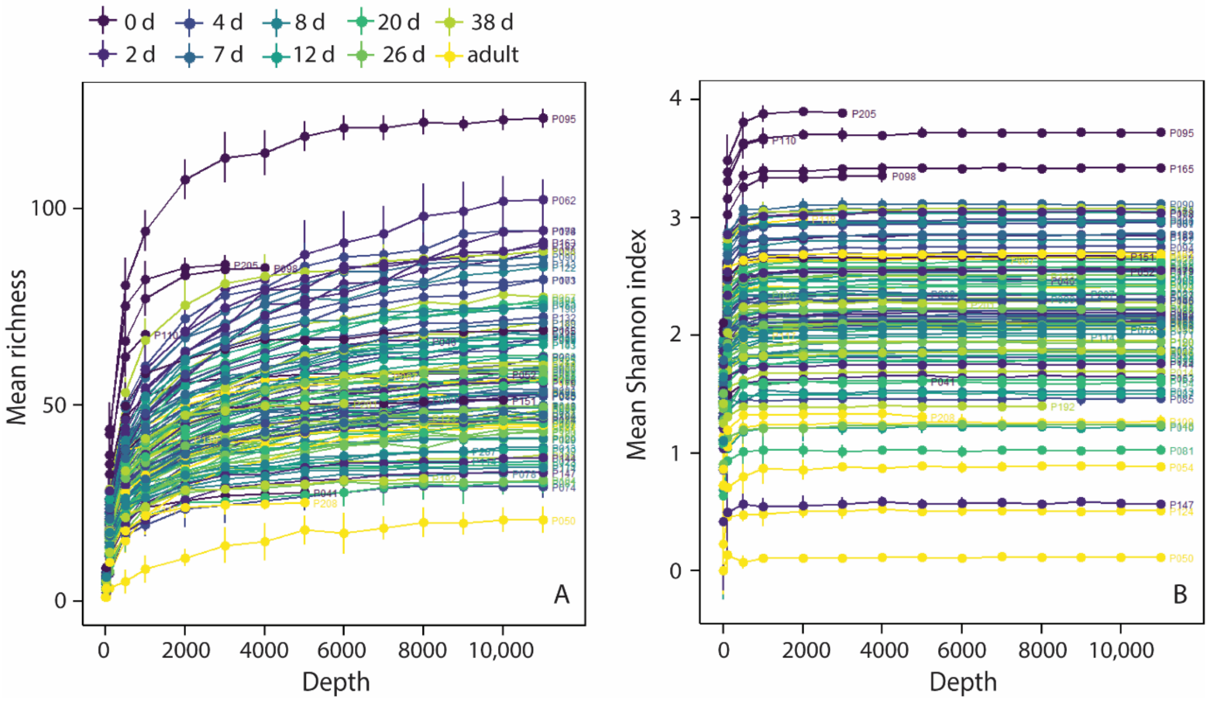

Figure S1. Rarefaction curves for richness (A) and Shannon index (B). Most curves levelled off around 4,000 reads. Sample sizes: per age per treatment group 6 chicks (3 nests), except for day 0 (4 normal food chicks and 3 food restricted chicks), and day 26 (6 normal food chicks and 5 food restricted chicks); and 11 adults.
